# Reversed-Phase UHPLC Enantiomeric Separation of Rasagiline Salts Using a Chiralpak^®^ AGP Column

**DOI:** 10.3390/scipharm85030026

**Published:** 2017-07-19

**Authors:** Nagarajan Balaji, Sayeeda Sultana

**Affiliations:** Department of Chemistry, St. Peter’s University, Avadi, Chennai 600 054, Tamil Nadu, India; sayeeda2006@yahoo.com

**Keywords:** chiral AGP, UHPLC, chiral separation, validation, rasagiline enantiomer

## Abstract

We report the first rapid ultra-high performance liquid chromatographic (UHPLC) enantiomeric reversed-phase separation of rasagiline mesylate and its tartrate salts using a Chiralpak^®^ AGP column (50 mm × 2.1 mm, 5 μm) as a stationary phase. This method was developed as an alternative to the usage of previously reported normal-phase chiral LC columns for isomer separation. Our method is based on an isocratic approach using a mixture of ammonium acetate and isopropyl alcohol (90:10, *v/v*) as the mobile phase (0.6 mL/min flow rate). The detection limit (at a detection wavelength of 210 nm) and quantification limit for the rasagiline enantiomers were 0.06 and 0.2 μg/mL, respectively. This method is compatible with the UHPLC-MS technique. The successful separation of rasagiline and its enantiomer was confirmed by determining the corresponding specific optical rotation values. Our method will be applicable for detecting rasagiline enantiomers during the control of manufacturing processes, and for use in rapid analysis for quality control in pharmaceutical industry to obtain optically pure pharmaceutical substances. This method was validated in terms of its precision, limit of detection, limit of quantification, linearity, accuracy, robustness, ruggedness, specificity, forced degradation, and solution stability, according to International Council on Harmonization Validation Guidelines Q2 (R1).

## 1. Introduction

**Figure 1:**
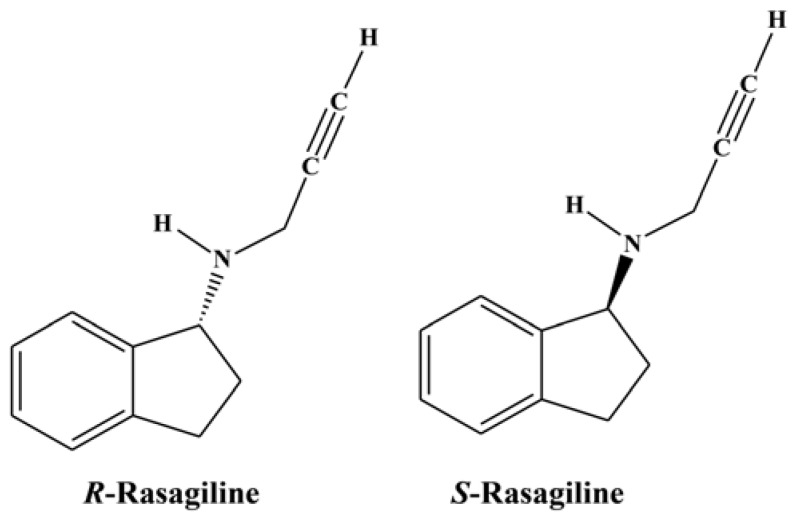
Chemical structures of *R*-rasagiline (RAS) and *S*-rasagiline (RAS-III).

Rasagiline mesylate is a pharmaceutical substance employed in the treatment of Parkinson’s disease, and its tartrate salt has displayed promise in terms of a cure for the disease [1,2,3,4,5,6,7]. Rasagiline mesylate itself has a single chiral center, resulting in the presence of two optical isomers (i.e., the *R*- and *S*-enantiomers, see Figure 1), with the *R*-form being pharmacologically active and the *S*-form being inactive [1,2,3,4,5,6,7]. Hence, in the manufacture of rasagiline, it is necessary to implement a strategy to control the formation of and/or detect the presence of the inactive *S*-enantiomer. This often involves the use of a specific and accurate liquid chromatographic (LC) method. Ideally, in the synthesis of rasagiline, the two isomers should be separated using chiral stationary phases. However, these isomers cannot be separated using C18, C8, or other stationary phases due to their comparable physicochemical properties. It is therefore necessary to either derivatize the isomers using chiral reagents, or employ a chiral stationary phase to separate the two enantiomers by LC techniques.

To date, the detection and quantification of rasagiline have been reported using a number of analytical techniques, including high-performance thin layer chromatography (HPTLC) [8], UV-visible spectroscopy [9,10] through the formation of colored ion pair complexes in acidic media [11], high-performance liquid chromatography (HPLC) with a range of mobile phase mixtures [12,13,14,15,16,17], and LC-MS/MS (mass spectrometry) estimations [18,19]. However, previous reports in this area describe the analysis of the rasagiline racemic mixture, and do not address the detection or quantification of the separate enantiomers. Nonetheless, later studies have reported the HPLC enantiomeric separation of these isomers using a polysaccharide-based Chiralcel OJ-H (Cellulose tris-(4-methylbenzoate)) column and a Chiralpak^®^ AD-RH (Amylose tris (3,5-dimethylphenylcarbamate)) column in the normal [20] and reversed-phase modes [21], respectively, in addition to a crown ether-based Chirosil RCA(+) ((+)-(18-crown-6)-tetracarboxylic acid) column in the polar organic mode [22].

In contrast, the use of chiral ultra-high performance liquid chromatography (UHPLC) for the separation of the rasagiline mesylate enantiomers and its tartrate salts has not yet been reported. We therefore selected this technique for our study, and employed a Chiralpak^®^ AGP (α1-acid glycoprotein) column for the first time as the stationary phase for the determination of these compounds.

## 2. Materials and Methods

### 2.1. Materials and Reagents

*R*- and *S*-Rasagiline mesylate and rasagiline tartrate were synthesized in the laboratory at St. Peter’s University, Avadi, Chennai, India. [1,2,3,4,5,6,7]. Ammonium acetate (99%) and isopropyl alcohol (99.5%) were purchased from Fisher Scientific (Mumbai, India). The Chiralpak^®^ AGP column (50 mm × 2.1 mm, 5 μm) was obtained from Daicel Chiral Technologies (Hyderabad, India). USP (United States pharmacopeia) grade water was employed throughout, and was prepared using a Metrohm Elga water purifier (Metrohm, Wycombe, United Kingdom). The 0.22 μm membrane filter paper and 0.22 μm syringe filters were obtained from Millipore (Bangalore, India).

### 2.2. Instrumentation

An Agilent Infinity LC 1290 (Germering, Germany) equipped with a binary bump with integrated vacuum degasser (G4220A), an autosampler (G4226A), a thermostatted column compartment (G1316C), and a diode array detector (G4212A) was used for the UHPLC separation of the rasagiline enantiomers. Thermal degradation studies were carried out using a hot air oven (MACK Pharmatech, Mumbai, India), while photodegradation studies were performed in a photostability chamber (Newtronics Photostability Chamber, Kandivali, Mumbai, India). An Autopol-II automatic polarimeter (Rudolph Research Analytical, Hackettstown, NJ, USA) was employed for determination of the specific optical rotations. Finally, a Shimadzu LC-8A preparative liquid chromatograph (Shimadzu, Chiyoda-ku, Tokyo, Japan) was utilized for isolation purposes.

### 2.3. Chromatographic Conditions

A Chiralpak^®^ AGP column with a length of 50 mm, an internal diameter of 2.1 mm, and a particle size of 5 μm was used as the stationary phase. The buffer solution was prepared by dissolving ammonium acetate (10 mmol/L) in water (1000 mL). The mobile phase was prepared by mixing the buffer solution (900 mL) with isopropyl alcohol (100 mL), followed by filtration through a 0.22 μm membrane filter. A flow rate of 0.6 mL/min was employed, along with a column temperature of 25 °C, a detection wavelength of 210 nm, and an injection volume of 2.0 μL. The conditions employed for preparative LC were as follows: The Chiralpak^®^ AGP column (150 mm × 10 mm, 5 μm) was used as the stationary phase. The buffer solution was prepared by dissolving ammonium acetate (10 mmol/L) in water (10,000 mL), and the mobile phase was prepared by mixing the buffer (9000 mL) with isopropyl alcohol (1000 mL), followed by filtration through a 0.22 μm membrane filter. A flow rate of 10 mL/min was employed in addition to a detection wavelength of 210 nm, an injection volume of 700 μL, and a column temperature of 25 °C. The sample solution was prepared by dissolving rasagiline mesylate (200 mg) in methanol (20 mL). The isolated solution was distilled by vacuum distillation, and separation was confirmed by determination of the specific optical rotation of each product.

### 2.4. Preparation of the Standard and Sample Solutions

The standard stock solution was prepared by dissolving the *S*-rasagiline enantiomer (3 mg) in water (100 mL). The standard solution was then prepared by diluting a portion of the standard stock solution (1 mL) to 100 mL with water. The concentration of the standard solution was 1.5 μg/mL with respect to the analyte concentration (i.e., rasagiline mesylate and its tartrate). The sample solution was prepared by dissolving rasagiline mesylate (20 mg) and rasagiline tartrate (20 mg) in water (100 mL). For preparation of the spiked sample solutions, the sample (20 mg) was dissolved in water (90 mL), and a portion of the standard stock solution (1 mL) was added and the solution made up to 100 mL with water (six preparations). All spiked samples, standards, and sample solutions were passed through a 0.22 μm syringe filter and introduced into the chromatograph using an autosampler. Direct injection of the sample solution was not suitable due to the increase in column back pressure, likely due to column clogging through the deposition of particles in the column pores. Approximately 10 batches of samples were analyzed.

### 2.5. Preparation of the Stress Study Solutions

**Figure 2:**
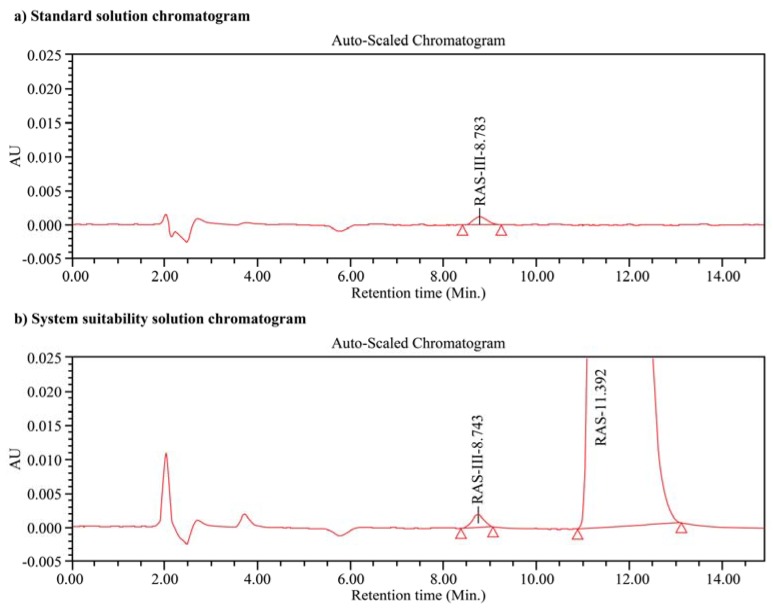
Chromatograms of (**a**) the standard and (**b**) the system suitability (RAS-III = *S*-enantiomer, RAS = *R*-enantiomer) solutions. Chromatographic conditions: Chiralpak^®^ AGP column (50 mm × 2.1 mm, 5 μm); 90:10 (*v/v*) mixture of ammonium acetate and isopropyl alcohol as the mobile phase; 0.6 mL/min flow rate; 210 nm detection wavelength; and 2.0 μL injection volume.

For the acid degradation experiments, the sample (20 mg) was dissolved in water (60 mL), and a 0.4 mol/L hydrochloric acid solution (20 mL) was added. This solution was maintained at 60 °C for 24 h in a water bath, after which time it was cooled to 25 °C, neutralized with a 1 mol/L sodium hydroxide solution (8 mL) (tested with pH paper), and diluted to 100 mL with water. For the basic degradation experiments, the sample (20 mg) was dissolved in water (60 mL), and a 0.4 mol/L sodium hydroxide solution (20 mL) was added. This solution was maintained at 60 °C for 24 h in a water bath, after which time it was cooled to 25 °C, neutralized with a 1 mol/L hydrochloric acid solution (8 mL) (tested with pH paper), and diluted to 100 mL with water. For the peroxide degradation experiments, the sample (20 mg) was dissolved in water (60 mL), and a 7.5% (*w/v*) hydrogen peroxide solution (40 mL) was added. This solution was maintained at 25 °C for 24 h. For the photodegradation experiments, the sample (20 mg) was dissolved in water (100 mL). The resulting solution was then placed in the photostability chamber and exposed to 1.2 million lux hours and 200 W·h/m^2^ radiation to promote photodegradation. For the thermal degradation process, a uniform distribution of the sample (5 g, maximum 5 mm thickness) was dried in a petri dish at 105 °C for 7 d in a hot air oven. After this time, the sample (20 mg) was dissolved in water (100 mL). The chromatograms of the standard and system suitability solutions are shown in Figure 2. The acid degradation, base degradation, peroxide degradation, thermal degradation, and photodegradation experiments are discussed in further detail in Section 3.8.

## 3. Results and Discussion

### 3.1. Method Development

In recent years, several trials have been performed involving the investigation of different chiral stationary phases for use in the separation of the two rasagiline enantiomers (Table 1) [19,20]. However, we selected the α1-acid glycoprotein (AGP) column as the chiral stationary phase for our study due to its unique properties. For example, this column can be utilized between pH 4 and 7, and can also be used in the reversed-phase mode [21,22].

As outlined in Table 1, the use of a normal phase mobile phase preparation was previously reported to lead to issues with quality control analysis, due to the increased probability of solvent evaporation and resulting variations in retention times and peak resolutions. To overcome this issue, we selected a reversed-phase mobile phase preparation, which differed from that previously suggested by Nirogi et al. [21]. In trial 1, the mobile phase was prepared as follows. Initially, a 10 mmol/L aqueous potassium phosphate solution was selected as the buffer and methanol was used as the solvent. The buffer-to-methanol ratio was varied as follows: 70:30, 80:20, 90:10, and 95:*5 (v/v*). However, no separation was observed between the isomers under these conditions. In trial 2, the buffer pH was adjusted to pH 6.0 using orthophosphoric acid, and buffer-to-methanol ratios of 70:30, 80:20, 90:10, and 95:5 (*v/v*) were examined. In this case, isomer separation was observed as a USP resolution of ~1.0 for a buffer-to-methanol ratio of 90:10. In trial 3, the solvent was changed from methanol to isopropyl alcohol, and buffer-to-isopropyl alcohol ratios of 70:30, 80:20, 90:10, and 95:5 (*v/v*) were examined. Although the USP resolution between isomers was ~2.0 for a buffer-to-isopropyl alcohol ratio of 90:10, the baseline was somewhat disturbed. In trial 4, ammonium acetate was used to prepare the buffer, as it has the added advantage of mass compatibility. Therefore, a 10 mmol/L aqueous ammonium acetate solution was used as the buffer, which was mixed in a 90:10 ratio with isopropyl alcohol. Using this mobile phase, the obtained USP resolution was >2.0, when compared to other trials. As described above, the preparation of our mobile phase was straightforward compared to previously reported methods, although it provided a comparable resolution between the two rasagiline enantiomers (i.e., ~2.9). However, the original run time for this method was rather long, and so we attempted to reduce it by increasing the column flow rate. Unfortunately, the column pressure increased to 300 bar, which was higher than the recommended operation conditions specified by the manufacturer [23,24]. We therefore adjusted the mobile phase composition to a 75:25 ratio of buffer to isopropyl alcohol, but this gave a poor resolution of 0.8. As stated in the literature, the minimum baseline resolution criterion between peaks for any analytical method is 1.5 [25]. The optimal conditions for UHPLC were therefore determined to be as follows: 10 mmol/L ammonium acetate in water (buffer), 90:10 ratio of ammonium acetate buffer to isopropyl alcohol as the mobile phase, 0.6 mL/min flow rate, 210 nm detection wavelength, 2.0 μL injection volume, and a Chiralpak AGP (50 mm × 2.1 mm) UHPLC column.

Initially, the same UHPLC chromatographic conditions were employed for the preparative LC process, and these conditions were found to be suitable for product isolation. Thus, using these optimized conditions on a preparative LC system equipped with a chiral preparative LC column, separation of the two rasagiline enantiomers was carried out. The final separation conditions for preparative LC were therefore as follows: 10 mL/min column flow rate, a mobile phase consisting of ammonium acetate buffer (900 mL of 10 mmol/L) and isopropyl alcohol (100 mL), 300 μL injection volume, 210 nm detection wavelength, and a 150 mm (length) × 10 mm (internal diameter) column with a 5 μm particle size. The fractions containing each individual isomer were concentrated and distilled under reduced pressure at 60 °C. The optical rotations of the resulting products were determined, and the successful separation of the two isomers was confirmed by the observation of two specific optical rotation values at +20° and −19°, corresponding to the *R*- and *S*-enantiomers, respectively (concentration = 0.6 mg/mL in water, temperature = 25 °C, wavelength = 589 nm). It should be noted that the reference value for the specific optical rotation of *R*-rasagiline is +21° [25], which is comparable to the obtained value. Two UHPLC columns were used for development and validation. The total number of injections in the development column was 300, and so this column was employed for the forced degradation study and intermediate precision analysis. A new UHPLC column was used for validation analysis. Different retention times were observed for the enantiomers using the development column due to the age of the column. However, a resolution between isomers of >1.5 was achieved. This was confirmed by MS measurements through observation of a species with the same mass as rasagiline rather than as the degradation products (as confirmed by the forced degradation and stress studies).

### 3.2. Analytical Method Validation

In terms of method validation, the system suitability and precision, limit of detection (LOD), limit of quantitation (LOQ), linearity and range, recovery, specificity, stress, robustness, and solution stability of this method for the analysis of the rasagiline enantiomers were determined as per the International Council on Harmonization (ICH) validation guidelines Q2, (R1) [26]. Further details regarding each of the above points can be found in the following subsections.

### 3.3. System Suitability and Precision

As previously mentioned, a separation resolution of 2.9 was established between the two rasagiline enantiomers upon injecting the system suitability solution into the chromatograph according to the optimized conditions. To determine the precision of this analytical system (i.e., an expression of the closeness of agreement between a series of measurements obtained from multiple sampling of the same homogeneous sample under the prescribed conditions) [26], the standard solution was injected into the chromatograph six times and the percentage relative standard deviation (% RSD) was calculated. The obtained % RSD of <3% indicates that this system was precise (% RSD limit = ≤5%) [27], and as such, is suitable for analysis of the rasagiline enantiomers. Further details regarding determination of the system precision are outlined in Table 2 below.

### 3.4. Method Precision

To determine the precision of this method, six spiked sample solutions were initially prepared. Using the above described method, the % RSD for the *S*-rasagiline content was 1.5% for the method precision (limit = <2%) [27], and was within 1.5% for the intermediate precision when performed by different analysts, on different columns and instruments on different days. These observations, in combination with the detailed results outlined in Table 3, indicate that our developed technique was suitably precise for the system of interest.

### 3.5. Limit of Detection and Limit of Quantification

The method detection limit (MDL) and method quantification limit (MQL) (i.e., the limit of detection, LOD, and limit of quantification, LOQ) were determined based on the signal to noise (S/N) ratio method as outlined in the ICH guideline Q2 (R1). Upon injecting the solution sequence of predetermined known concentrations (0.2–2.2 μg/mL), the S/N ratio for the LOD was determined to be 3:1, while that of the LOQ was determined to be 10:1. Thus, the MDL and MQL for *S*-rasagiline were 0.06 and 0.20 μg/mL, respectively, which were very low when compared to those of references [20,21]. These results indicate that the method was sufficiently sensitive for determination of the *S*-enantiomer content in the sample of rasagiline mesylate and its tartrate.

### 3.6. Linearity and Range

**Figure 3:**
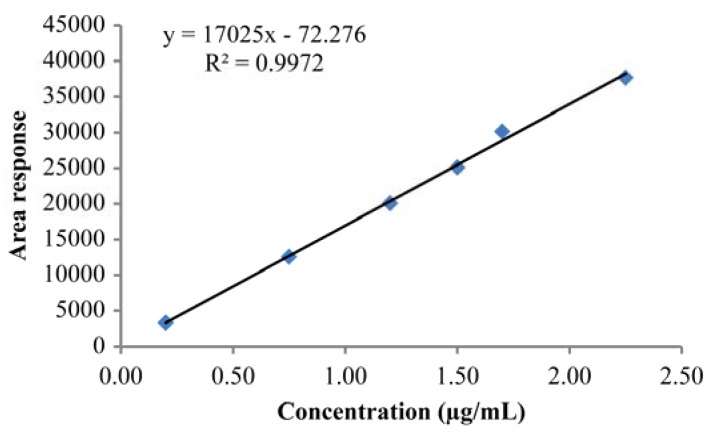
Linearity plot for the *S*-enantiomer (RAS-III).

The linearity of an analytical procedure reflects its ability to produce results that are directly proportional to the concentration of an analyte in the sample [26]. In this case, linearity tests were performed from the LOQ to 150% of this limit for an analyte concentration of 200 μg/mL. The results of this test and the corresponding correlation coefficient are shown in Table 4, while the linearity plot is provided in Figure 3. As shown, the correlation coefficient was close to 1, indicating that the developed method was indeed linear. Furthermore, the statistical linear regression results indicate that the validated method was linear for the rasagiline system, and that this linearity was satisfactory over the defined concentration range (i.e., 0.2–2.2 μg/mL).

### 3.7. Accuracy

The accuracy of an analytical procedure indicates the closeness of understanding between the quality, which is acknowledged either as a conventional true value or an accepted reference value, and the observed value [26]. For quantitative approaches, at least nine determinations across a specified range should be obtained [26]. In our case, the accuracies (%) for detecting the *S*-enantiomer in separate mixtures of rasagiline mesylate and its tartrate were 95–100% and 91–100%, respectively. These results indicate that our developed method was accurate for the present analytical system, as the mean accuracy value was within the standard 80–120% limit. Furthermore, the accuracies of this method at the LOQ and at 50, 100, and 150% levels of the LOQ for the *S*-enantiomer are outlined in Table 5 and Table 6.

### 3.8. Specificity and Stability Studies

**Figure 4:**
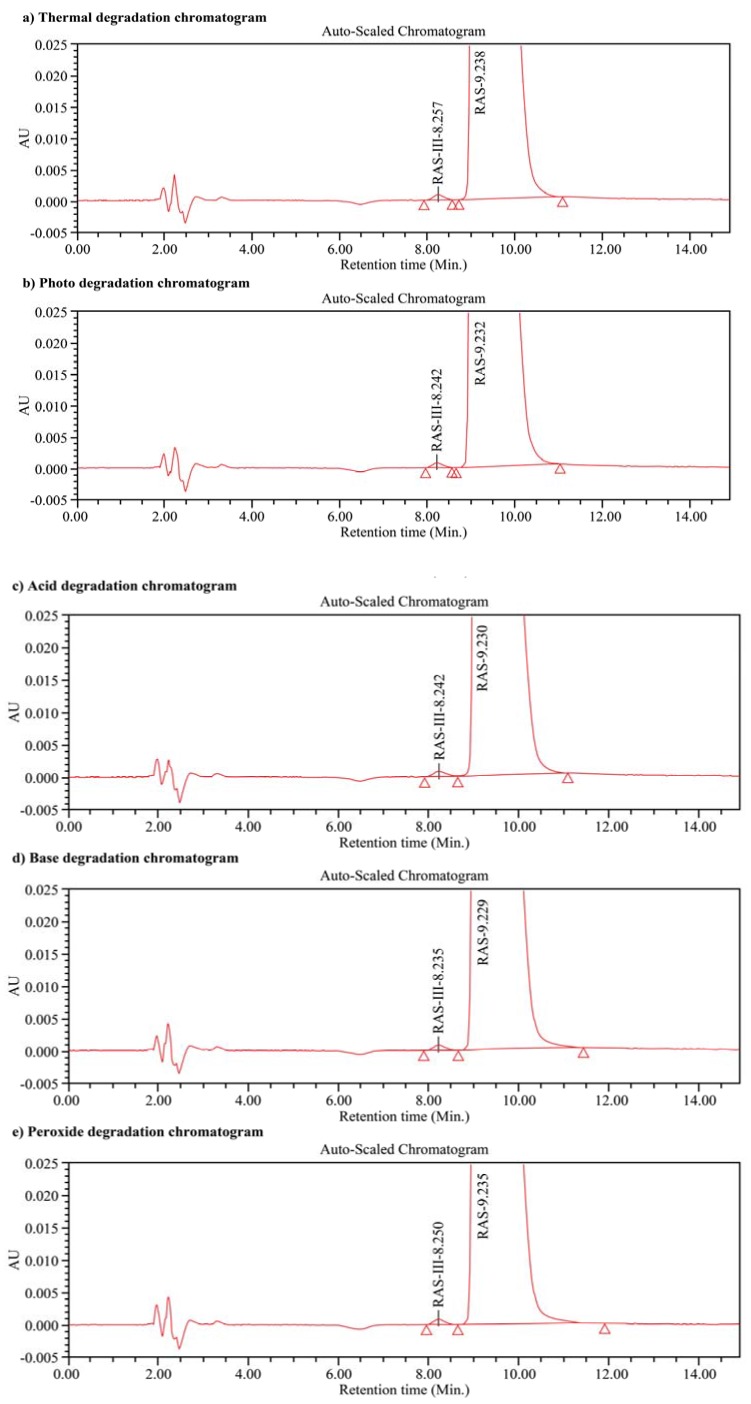
Chromatograms of (**a**) the acid degraded; (**b**) the base degraded; (**c**) the peroxide degraded; (**d**) the thermally degraded; and (**e**) the photodegraded solutions. RAS-III = *S*-enantiomer, RAS = *R*-enantiomer. Chromatographic conditions: Chiralpak^®^ AGP column (50 mm × 2.1 mm, 5 μm); 90:10 (*v/v*) mixture of ammonium acetate and isopropyl alcohol as the mobile phase; 0.6 mL/min flow rate; 210 nm detection wavelength; and 2.0 μL injection volume.

Specificity is the ability to assess the analyte unequivocally in the presence of other components that may be present in the mixture. These might typically include impurities, degradants, and matrix components, among others [26]. Thus, the specificity of our method was determined by examination of the peak purity (i.e., the purity angle should be lower than the purity threshold) using a photodiode array detector for the forced degradation samples. To confirm the specificity of our system, all forced degradation samples examined were investigated using a sample concentration of 200 μg/mL. For both the *R*- and *S*-rasagiline enantiomers, no interference was observed either from the blank or from impurities. In addition, no secondary peaks originating from degraded species were observed. Furthermore, the results of the forced degradation study indicate that the *S*-rasagiline was not a degradation impurity. The chromatograms of the acid degraded, base degraded, peroxide degraded, thermally degraded, and photodegraded solutions are shown in Figure 4. These results confirm the specificity/homogeneity of our developed method for the detection of *S*-rasagiline.

### 3.9. Robustness

We then examined the effect of chromatographic conditions on the resolution between the two rasagiline enantiomers. As the original mobile phase flow rate was 0.6 mL/min, we varied the flow rate from 0.5 to 0.7 mL/min to investigate its effect on the resolution. In addition, the column oven temperature was set at 20 °C, 25 °C, or 30 °C to examine the effect of temperature. Finally, the isopropyl alcohol content in the mobile phase was varied between 80 mL and 120 mL at 20 mL intervals. Interestingly, the resolution was >1.8 under all conditions studied, thus demonstrating the robustness of our method.

### 3.10. Solution Stability

Finally, the solution stability was determined by examination of a freshly prepared standard solution and a sample solution stored in a sealed volumetric flask at 25 °C over 24 h. The % difference between the peak areas of *S*-rasagiline at 0 and 24 h was <15.0 for the standard solution, and the % difference between the contents of *S*-rasagiline at 0 and 24 h was <15.0 for the sample solution [26]. These results therefore indicate that the enantiomer solution was stable under the above conditions.

## 4. Conclusions

We herein reported the use of a volatile mobile phase compatible with mass spectrometry (i.e., ammonium acetate and isopropyl alcohol in water) in a rapid chiral reversed-phase ultra-high performance liquid chromatographic (UHPLC) method for the determination and separation of the pharmaceutically inactive *S*-enantiomer of rasagiline mesylate from a mixture of the two enantiomers and rasagiline tartrate. The final separation conditions were as follows. A Chiralpak^®^ AGP column was used as the stationary phase, and a mixture of 10 mmol/L ammonium acetate and isopropyl alcohol was employed as the mobile phase. The column oven temperature was 25 °C, the injection volume was 2.0 μL, and the detection wavelength was 210 nm. More specifically, a Chiralpak^®^ AGP column was employed in our precise and accurate method, yielding acceptable and repeatable recoveries in addition to low limits of detection and quantification. The successful separation of the enantiomers was confirmed by optical rotation measurements, which were confirmed by comparison with literature values. This authenticated method is expected to be applicable in the regular analysis of rasagiline mesylate enantiomers in quality control laboratories during the preparation of this pharmaceutical agent. However, further studies are required to decrease the run time of our method, as this was not possible through simply increasing the column flow rate.

## Figures and Tables

**Table 1:** Comparison of the proposed method with reported methods for the separation of rasagiline enantiomers.

Method	Column	Resolution	LOD; LOQ *	Run Time	Reference
HPLC	Chiralcel OJ-H 250 × 4.6 mm;	5.4	0.35 μg/mL; 1.05 μg/mL	Requires more time to stabilize the LC system.	[20]
Normal phase (n-hexane/isopropyl alcohol/ethanol/diethyl amine) (96:2:2:0.01, *v/v/v/v*)				Run time: 20 min.	
HPLC	Chiralpak AD-RH 150 × 4.6 mm;	3.5	0.16 μg/mL; 0.49 μg/mL	Requires less time to stabilize the LC system compared to method [21].	[21]
Reversed-phase (20 mmol/L potassium dihydrogen phosphate in water/acetonitrile (65:35, *v/v*) adjusted to pH 6.9 using 10 wt % potassium hydroxide)				Run time: 25 min.	
HPLC	Chirosil RCA(+) 250 × 4.6 mm;	>2.0	Not available	~20 min.	[22]
Polar organic phase (ethanol/acetonitrile/acetic acid/triethylamine (80:20:0.2:0.3, *v/v/v/v*))					
UHPLC	Chiralpak^®^ AGP 50 × 2.0 mm	2.9	0.06 μg/mL; 0.20 μg/mL	MS-compatible, rapid analysis, easy to stabilize the LC system compared to other normal phase [20] and reversed-phase [21] methods.	Present work
Reversed phase (ammonium acetate/isopropyl alcohol (90:10, *v/v*))				Run time: 15 min.	

* LOD: Limit of detection; LOQ: Limit of quantification; UHPLC: ultra-high performance liquid chromatography.

**Table 2:** Determination of the system suitability and precision.

**% RSD of Peak Area**		
**Inj. No.**	***S*-Rasagiline (RAS-III) (1.5 μg/mL)**	**Criteria**
1	24,865	≤5%
2	24,510	
3	24,279	
4	23,787	
5	22,957	
6	24,881	
Mean	24,213	
SD	738	
% RSD	3.0	
**Resolution between RAS-III and *R*-Rasagiline (RAS)**		
**Inj. No.**	**Resolution**	**Criteria**
1	2.9	≥1.5
**K Prime (Retention Factor)**		
**Inj. No.**	***S*-Rasagiline (RAS-III)**	***S*-Rasagiline (RAS-III) Repeatability of K**
1	8.9	8.8
**Peak Symmetry Factor**		
**Inj. No.**	***S*-Rasagiline (RAS-III)**	***R*-Rasagiline (RAS)**
1	1.0	1.1
**Inj. No.**	**Selectivity**	
1	2.9	

RSD: relative standard deviation.

**Table 3:** Method and intermediate precision results for *S*-rasagiline (RAS-III).

Preparation No.	RAS-III Content (1.5 μg/mL)	RAS-III Content (1.5 μg/mL)
	Method Precision	Intermediate Precision
1	0.145	0.151
2	0.147	0.152
3	0.149	0.150
4	0.150	0.153
5	0.150	0.149
6	0.151	0.148
Mean	0.148	0.150
% RSD	1.5	1.2

**Table 4:** Linearity data for the *S*-enantiomer (RAS-III).

Sample No.	% Level	Concentration (μg/mL)	Peak Response
1	LOQ	0.20	3349.00
2	50	0.75	12,560.50
3	80	1.20	20,096.80
4	100	1.50	25,121.00
5	120	1.70	30,145.20
6	150	2.25	37,681.50

**Table 5:** Accuracy of *S*-enantiomer detection in the rasagiline mesylate mixture at various concentration levels.

Accuracy Level	Spiked Amount (wt %)	Mean Content (wt %)	Content in Spiked Sample (wt %)	Recovered Content (wt)	% Recovery	Mean % Recovery	% RSD
LOQ-1	0.003	0.004	0.007	0.003	100.000	100.00	0.00
LOQ-2	0.003		0.007	0.003	100.000		
LOQ-3	0.003		0.007	0.003	100.000		
50%-1	0.074	0.004	0.075	0.071	95.945	95.93	0.03
50%-2	0.073		0.074	0.070	95.890		
50%-3	0.074		0.075	0.071	95.945		
100%-1	0.147	0.004	0.144	0.140	95.238	95.48	0.40
100%-2	0.147		0.145	0.141	95.918		
100%-3	0.148		0.145	0.141	95.270		
150%-1	0.222	0.004	0.215	0.211	95.045	95.19	1.18
150%-2	0.221		0.217	0.213	96.380		
150%-3	0.222		0.213	0.209	94.144		

RSD: relative standard deviation; wt: weight.

**Table 6:** Accuracy of *S*-enantiomer detection in rasagiline tartrate at various concentration levels.

Accuracy Level	Spiked Amount (wt %)	Mean Content (wt %)	Content in Spiked Sample (wt %)	Recovered Content (wt)	% Recovery	Mean % Recovery	% RSD
LOQ-1	0.004	0.001	0.005	0.004	100.000	100.0	0.00
LOQ-2	0.004		0.005	0.004	100.000		
LOQ-3	0.004		0.005	0.004	100.000		
50%-1	0.072	0.001	0.067	0.066	91.666	91.59	0.07
50%-2	0.071		0.066	0.065	91.549		
50%-3	0.071		0.066	0.065	91.549		
100%-1	0.143	0.001	0.135	0.134	93.706	93.47	0.43
100%-2	0.143		0.134	0.133	93.006		
100%-3	0.143		0.135	0.134	93.706		
150%-1	0.215	0.001	0.198	0.197	91.627	92.24	1.60
150%-2	0.214		0.202	0.201	93.925		
150%-3	0.215		0.197	0.196	91.162		
